# Maturity of white matter tracts is associated with episodic memory recall during development

**DOI:** 10.1093/texcom/tgac004

**Published:** 2022-01-27

**Authors:** Antoine Bouyeure, Dhaif Bekha, Sandesh Patil, Lucie Hertz-Pannier, Marion Noulhiane

**Affiliations:** 1 UNIACT, NeuroSpin, CEA, Université Paris-Saclay, 91191 Gif-sur-Yvette, France; 2 UMR1141, Inserm, Université de Paris, 75019 Paris, France

**Keywords:** diffusion weighted imaging, episodic memory, memory development, white matter

## Abstract

The structure-function relationship between white matter microstructure and episodic memory (EM) has been poorly studied in the developing brain, particularly in early childhood. Previous studies in adolescents and adults have shown that episodic memory recall is associated with prefrontal-limbic white matter microstructure. It is unknown whether this association is also observed during early ontogeny. Here, we investigated the association between prefrontal-limbic tract microstructure and EM performance in a cross-sectional sample of children aged 4 to 12 years. We used a multivariate partial least squares correlation approach to extract tract-specific latent variables representing shared information between age and diffusion parameters describing tract microstructure. Individual projections onto these latent variables describe patterns of interindividual differences in tract maturation that can be interpreted as scores of white matter tract microstructural maturity. Using these estimates of microstructural maturity, we showed that maturity scores of the uncinate fasciculus and dorsal cingulum bundle correlated with distinct measures of EM recall. Furthermore, the association between tract maturity scores and EM recall was comparable between younger and older children. Our results provide new evidence on the relation between white matter maturity and EM performance during development.

## Introduction

The maturation of the microstructural properties of white matter pathways is pivotal to cognitive development. Many cognitive functions such as working memory, language, or theory of mind, have been shown to be associated to the integrity of white matter tracts in the adult and developing brains (Nagy et al. 2004; Krogsrud et al. 2018; Mürner-Lavanchy et al. 2018; Walton et al. 2018; Short et al. 2019). This is also the case for episodic memory (EM), the ability to remember events that occurred in a given spatiotemporal context over long-term delays. Several studies demonstrated a structural–functional relationship between white matter integrity and EM performance during development (Mabbott et al. 2009; Schaeffer et al. 2014; Samara et al. 2019). However, because this association has mainly been examined in preadolescents or adolescents, the contribution of white matter maturation to the ontogeny of EM during early childhood is poorly known.

This paucity of data is damageable because the first 7 years of life are a period during which EM abilities increase greatly. This period also corresponds to infantile and childhood amnesia, which are defined by an absence, at adult age, of episodic memories from early childhood (Newcombe et al. 2007; Picard et al. 2012; Guillery-Girard et al. 2013; Lavenex and Banta Lavenex 2013; Olson and Newcombe 2013; Bauer 2015; Bouyeure et al. 2020; Bouyeure and Noulhiane 2021). Development of EM during childhood has been related to the structural and functional maturation of the hippocampus (e.g. Newcombe et al. 2007; Lavenex and Banta Lavenex 2013; Olson and Newcombe 2013). How the maturation of white matter tracts contribute to the development of EM abilities in young children and during subsequent development remains to be further described. We studied the relationship between white matter maturity and EM recall in a cross-sectional sample of children aged from 4 to 12 years. We estimated white matter maturity using multivariate associations between measurements of white matter microstructure and age. We used these statistical approximations of white matter maturity to describe their associations with EM performance during development.

### Development of episodic memory

The development of EM is protracted and extends from early infancy to adolescence. The first two to three years of life are a period of rapid development of EM abilities, which has been associated to the structural and functional maturation of its cerebral substrates. Infants in their first year of life display rudimentary conscious long-term memory abilities (explicit memory), as deferred imitation paradigms showed that 6 months old infants can reproduce a learned sequence of actions over delays up to a month (Carver et al. 2000; Carver and Bauer 2001; Bauer et al. 2011; Bouyeure et al. 2020). These explicit memories have been shown to be mainly supported by cerebral activity in the medial temporal lobe region (Lavenex and Banta Lavenex 2013; Olson and Newcombe 2013; Mullally and Maguire 2014; Gómez and Edgin 2016; Alberini and Travaglia 2017), e.g. of the hippocampus and the parahippocampal cortices. Indeed, the hippocampus participates actively in the formation of early memories in infants (e.g. see Li et al. 2014; Travaglia et al. 2016; Alberini and Travaglia 2017) but is still largely immature in terms of structural and functional features. The size of the hippocampus nearly doubles during the first two years of life, reaching adult size around age 3 (Utsunomiya et al. 1999; Olson and Newcombe 2013).

From age 3 to 7, consequential albeit less dramatic structural and functional changes are observed in the hippocampus. While its overall size stays relatively stable, distinct patterns of volumetric changes are observed in the hippocampal subfields (Krogsrud et al. 2014; Daugherty et al. 2016; Canada et al. 2018; Riggins et al. 2018). These have been associated to the rapid increase of EM abilities during early childhood (Olson and Newcombe 2013; Riggins 2014; Riggins et al. 2015, 2016, 2018; Ngo et al. 2017; Canada et al. 2018). While the maturation of the hippocampus continues at a slower rate afterwards (Olson and Newcombe 2013), age-related increases of EM competence after age 7 have also been related to the development of neocortical regions. For example, increase of prefrontal activity and increase of prefrontal–hippocampal connectivity with age have been shown to correlate with EM performance (Menon et al. 2005; Chiu et al. 2006; McAuley et al. 2007; Ofen et al. 2007; Maril et al. 2010; Ghetti and Bunge 2012; Qin et al. 2014). Neocortical regions such as the prefrontal cortex thus seem to have an increasingly more important role in EM with developmental time, which could be related to an increasing demand on recall strategies and mnemonic control (for a discussion, see Ghetti and Bunge 2012). The maturation of white matter pathways connecting the limbic region (comprising the hippocampus) with the prefrontal cortex should therefore play a key role in the development of EM. It is however unknown if this contribution is to be found early during development or only emerges progressively.

### White matter correlates of episodic memory

The integrity of prefrontal–limbic pathways has been consistently associated with EM performance in adolescents and adults (Mabbott et al. 2009; Ghetti and Bunge 2012; Ngo et al. 2017; Samara et al. 2019). Major prefrontal–limbic tracts are the uncinate fasciculus (UF), the cingulum bundle (CB), and the fornix (Daitz and Powell 1954; Douet and Chang 2015; Olson et al. 2015; Bubb et al. 2018). The UF connects the prefrontal cortex and the anterior temporal lobe and is involved in episodic recall and mnemonic control (Olson et al. 2015; Wendelken et al. 2015). The CB connects the prefrontal cortex to the posterior medial temporal lobe by running along the cingulate gyrus, connecting the prefrontal cortex and subcortical nuclei and is associated with mnemonic control and EM recall and recognition (Zhuang et al. 2012; Ezzati et al. 2016; Bubb et al. 2018). It is often subdivided in functionally distinct segments (Bubb et al. 2018). The dorsal CB connects the prefrontal and parietal cortices and is associated with cognitive control, and the ventral CB runs along the medial temporal lobe and continues dorsally until the retrosplenial cortex and the parietal cortex and is associated with EM. Finally, the fornix connects the bilateral hippocampi to subcortical nuclei and the orbitofrontal cortex. Fornix microstructure has been shown to be critical to EM function in normal and pathological populations (Gaffan 1992; Zhuang et al. 2012; Douet and Chang 2015; Hodgetts et al. 2017).

The UF and the CB have a protracted maturation, lasting until early adulthood (Lebel et al. 2012; Lebel and Deoni 2018; Reynolds et al. 2019). In contrast, the Fornix is an early-maturing tract, showing adult-like microstructural properties during early childhood (Dubois et al. 2012; Lebel et al. 2012; Reynolds et al. 2019). Prefrontal–limbic tracts could have distinct contributions to EM function during development because of their different developmental trajectories. Previous studies have shown an association between prefrontal–limbic tract microstructure and EM in school-age children and adolescents (Mabbott et al. 2009; Schaeffer et al. 2014; Samara et al. 2019). The only study that examined white matter correlates of EM in young children (Ngo et al. 2017: 4–6 years) found no relation between prefrontal–limbic tracts and EM, indicating that the contribution of prefrontal–limbic connectivity (mediated by the UF) to EM might appear during later development. However, an examination of the relationship between the maturation of the prefrontal–limbic tracts and the development of EM covering different developmental periods still lacks to this date.

### The current study: a multivariate assessment of white matter–EM relations

Our aim was to examine the relation between white matter integrity and the development of EM during childhood. EM was assessed with the children’s version of the California Verbal Learning Tool (CVLT-c) (Delis 1994), which is widely used in both clinical and research contexts as it allows to measure distinct aspects of EM retrieval. We studied prefrontal–limbic tracts that have previously been associated with EM, namely the UF, the CB (ventral CB and dorsal CB), and the fornix.

The microstructure of white matter tracts is often examined with diffusion tensor imaging (DTI) parameters such as fractional anisotropy (FA), axial diffusivity (AD), and radial diffusivity (RD). The interpretation of these parameters provides distinct microstructural information about a structure (i.e. a given white matter tract). When they describe the same structure, they can be collinear to each other. For example, changes of FA value of a tract can derive from change of its AD, RD, or both (Wheeler-Kingshott and Cercignani 2009; Winklewski et al. 2018). Developmental studies typically assess the age-related differences and relationships with cognitive functioning of each diffusion parameter individually; for example, associations of EM with FA and mean diffusivity (a linear combination of AD and RD) have been reported (Mabbott et al. 2009). This type of approach has several advantages (e.g. relative straightforwardness of interpretation) but neglects the fact that individual differences of a cognitive function can be related to a pattern of differences within distinct properties of a given white matter tract. Age-related differences of white matter microstructure are likely to be related in distinct but linked ways to age-related differences of cognitive function. Moreover, another inconvenience of the classical approach is that the bivariate exploration of structure–function relationships for several diffusion parameters of several white matter tracts can lead to an inflation of the number of statistical tests required.

An interesting alternative is to use a multivariate statistical perspective. Specifically, partial least square correlation (PLSC) is a dimensionality reduction technique that has gained popularity in the field of neuroimaging in the recent years to study structure–function relationships (Abdi and Williams 2013; Chen et al. 2019; Garthwaite 1994; Keresztes et al. 2017; Krishnan et al. 2011; Van Roon et al. 2014). PLSC is designed to search for latent variables that represent the shared information between two sets of variables (e.g. brain features and behavior). In particular, this approach was recently used to study the relationship between memory discrimination and hippocampal maturity defined as the multivariate representation of the shared information between hippocampal subfields’ volumes and age (Keresztes et al. 2017). A similar approach could be fruitfully applied to the question of the relationship between white matter maturity and EM development, which we aimed to do here.

Our aim was threefold: (i) to determine if there was a multivariate pattern associating differences of tract microstructure with differences of EM performance during childhood. We hypothesized that the shared information between tract microstructure and measures of EM recall could be significantly represented by latent variables, demonstrating a multidimensional structure–function relationship. (ii) To determine if these multidimensional associations were specifically related to individual differences in tract maturity. We hypothesized that for tracts with a protracted maturation (e.g. the UF and the CB) the multivariate association described in step 1 could be specifically described by the relationship between individual differences in tract microstructural maturity and individual differences in EM abilities. Tract maturity was defined with PLSC by latent variables representing the shared information between tract microstructure and age (see Keresztes et al. 2017). We then correlated these estimations of tract maturity with EM scores. (iii) To determine if the relationship between tract maturity and EM performance was different between younger and older children. We hypothesized that these relations could differ as a function of age: for example, tracts with a protracted maturation could be too immature in younger children for their microstructural maturity to be associated as much with EM performance as in older children (see Ngo et al. 2017).

## Materials and methods

### Participants

We recruited 50 healthy children (22 females) aged 4 to 12 years old (mean age = 8.1, SD = 2.28). Ethical agreements were obtained from the appropriate ethical board and written consent of the children and their parents was collected. Participants completed a range of cognitive tasks and underwent a 45 min MRI protocol as part of a larger study on the maturation of the neural substrates of EM during childhood. Among our 50 participants, 11 had no data, had incomplete data, or were excluded because of a history of learning disorders or of structural anomalies detected on the MR images. Moreover, two participants were excluded because of low compliance during behavioral assessments. Therefore, we studied 37 children (15 females).

### Assessment of verbal episodic memory

Memory performance was assessed with the French adaptation of the children’s version of the CVLT-c (Delis 1994). The CVLT-c is widely used in clinical and experimental settings to assess verbal learning and verbal EM. It provides several verbal EM scores, including recall (free and cued) and a recognition test, with and/or without delay. The procedure of the CVLT-c is as follows: first, participants learn a list of 15 words belonging to 3 semantic categories through 5 learning trials. In each learning trial, the experimenter reads the list of words to the participant, who tries to recall the most words immediately afterward. A second list of words is then read to the participant followed by the recall of this second list. The participant is then asked to recall the words from the first list (the short-delay free recall score used in this study). A cued recall is also administered for each semantic category. After a 20 min delay, the participant is asked to recall words from the first list (long-delay free recall score used in this study). This is followed by another cued recall of the first list (long-delay cued recall used in this study) and a word recognition task. Given the multiplicity of indices provided by the CVLT-c, we chose to focus on three scores: short-delay free recall, long-delay free recall, and long-delay cued recall. These tests were chosen as they allow us to contrast two types of conditions: free recall (short-delay or long-delay) and delay recall (free or cued). We can thus examine distinct but related aspects of EM recall. This guarantees that the multivariate relationships studied here include conceptually related aspects of EM, which is easier for interpretation.

### MRI data acquisition

Imaging data was collected at the NeuroSpin research center, CEA, Gif-sur-Yvette, France. Children first followed an MRI training session on a mock scanner set in a children-friendly environment. They were told a compelling story, making them astronauts on a mission to understand the brain, taking aboard a spaceship (the scanner), and wearing a space helmet (the head coil). For the mission to succeed, children were told to try staying still as much as possible for the scanner to take accurate pictures of their brains. Once the children were familiarized with the sonic and visual environment of the scanner, the acquisition began. Images were acquired on a Siemens PRISMA 3 T scanner (Siemens Medical Solutions, Erlangen, Germany) with a 64-channel head coil. The animation movie *Wall-E* (Pixar Animation Studios) was shown to children during the scanning sessions to bolster engagement and reduce head motion caused by intolerance to noise and the sensation of boredom.

We collected a standard high-resolution T1-weighted MPRAGE sequence of 160 axial slices (TR = 2.3 s, TE = 3.05 ms, FOV = 256 mm, 73° flip angle, 0.9 mm isotropic resolution). Multishell high-angular resolution diffusion-weighted data was acquired through two distinct sequences: (i) a sequence with a gradient *b*-value of 1,500 s/mm^2^ applied along 64 isotropic directions and (ii) a sequence with a gradient *b*-value of 1,000 s/mm^2^ applied along 30 anisotropic directions. Both sequences shared the following parameters: TE = 55.2 ms; voxel dimensions = 1.8 × 1.8 × 1.8mm^3^; field of view = 238 mm; 78 contiguous slices acquired along an axial plane with 1.8 mm thickness (no gap) in the posterior–anterior direction. For distortion correction, we collected 5 non–diffusion weighted images with *b* = 0 s/mm^2^ at the beginning of each sequence (same phase-encoding direction) and 5 non–diffusion weighted *b* = 0 s/mm^2^ images in the opposite phase-encoding directions.

### MRI Preprocessing

Individual *T*_1_-weighted images were segmented into gray matter (GM), white matter (WM), corticospinal fluid (CSF) tissue types while correcting for spatial intensity variations (*b*1 bias field) using FMRIB's Automated Segmentation Tool (FAST). The bias-corrected *T*_1_ images were further processed with the FreeSurfer anatomical pipeline to obtain cortical parcellations (Destrieux et al. 2010). For segmentation of subcortical structures, we chose to use FSL instead of FreeSurfer, as the latter is known to produce more “blocky” segmentations. Subcortical structures (including the hippocampus) were thus segmented with FMRIB's Integrated Registration & Segmentation Tool (FIRST; (Patenaude et al. 2011). We combined the resulting FreeSurfer cortical parcellations and FSL’s subcortical segmentations to produce individual cortical–subcortical maps. These were registered to the average *b*0 images of each subject with FSL’s Boundary-Based Registration Algorithm (Greve and Fischl 2009).

**Fig. 1:**
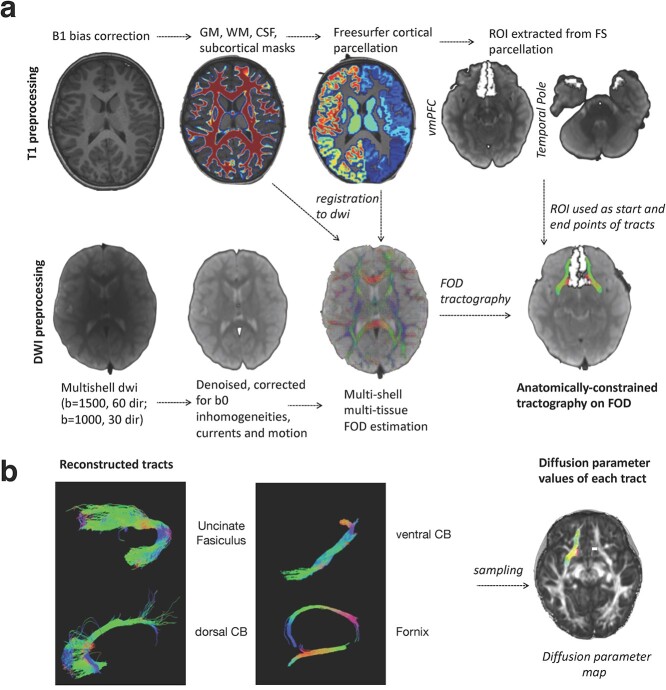
Overall analysis pipeline used in this study. a) Tract reconstruction pipeline. b) Examples of reconstructed tracts and sampling of diffusion parameters. GM = gray matter. WM = white matter. CSF = corticospinal fluid. FS = FreeSurfer. dwi = diffusion-weighted imaging. dir = directions. FOD = fiber orientation distribution. CB = cingulum bundle.

Diffusion data were preprocessed using a combination of tools from FSL (Jenkinson et al. 2012), version 6.0.0, http://fsl.fmrib.ox.ac.uk, and MRtrix3 RC3 (Tournier et al. 2019), https://www.mrtrix.org. We registered the average image of the b1000 sequence to the average image of the b1500 sequence with the Boundary-Based Registration Algorithm (Greve and Fischl 2009) as implemented in FSL (*epi_reg*). We concatenated the co-registered DWI sequences, obtaining a single multishell image including 2 *b*-values (1500 and 1000) for each subject. We then performed data denoising with MRtrix’s *dwidenoise* function, which exploits data redundancy in the PCA domain (Veraart et al. 2016), and removed Gibbs ringing artifacts (MRtrix’s *dwidegibbs*) (Kellner et al. 2016). We estimated the susceptibility-induced off-resonance field in the data using FSL *topup* (Smith et al. 2004). The resulting denoised, unringed, susceptibility-corrected images were corrected for eddy currents and motion with FSL *eddy* (Andersson and Sotiropoulos 2016). The corrupted slices were re-interpolated using a Gaussian process method implemented in the FSL *eddy* function (Andersson et al. 2016). Finally, we corrected the data for the B1 bias field with MRtrix *dwibiascorrect*. The general outline of our preprocessing pipeline is represented Fig. 1.

### Tractography

Probabilistic tractography of the selected tracts of interest was performed at the individual level using constrained spherical deconvolution (CSD; Tournier et al. 2007) as implemented in MRtrix. Tissue-specific (WM, GM, CSF) response functions were estimated with the multishell multitissue algorithm to estimate a “representative” fiber orientation density function (fODF) for each tissue type. These response functions were used as kernels by a CSD algorithm with default parameters to estimate continuous fODF at the voxel level. Anatomically constrained probabilistic tractography (Smith et al. 2012) was then performed on FOD peaks with anatomical priors (the WM, GM, and CSF maps) to constrain tractography within white matter. Cortical or subcortical ROIs were used as seeds and target regions to define specific tracts. The CB was subdivided in ventral and dorsal segments, with the cingulate isthmus as the demarcation point between the two segments (Bubb et al. 2018). Appropriate ROIs were selected for each tract based on prior works (UF: Olson et al. 2015; CB: Bubb et al. 2018; Fornix: Daitz and Powell 1954, Douet and Chang 2015). These tracts were selected based on their association to EM performance in previous studies conducted in children or adults (Mabbott et al. 2009; Schaeffer et al. 2014; Wendelken et al. 2015; Ngo et al. 2017; Samara et al. 2019). Streamlines were selected if they connected the selected ROIs at the location of their respective GM/WM interface while following a path along with FOD amplitude above 0.1 within the white matter mask. The seed ROIs were dilated in all directions by a factor of one voxel to allow the intersection between the ROI and the gray matter/white matter interface. Appropriate exclusion ROIs were additionally used to further guide tractography (Table 1). We used the “stop” option to stop the propagation of streamlines once they traversed all inclusion regions. Other parameters were set to default (theta angle: 45; min length: 5^*^voxel size; max length: 100^*^voxel size; trials number: 1000). For the fornix, we used modified anatomical masks as priors. The body of the fornix was not fully included in the original white matter masks because of contamination/partial volume effects resulting from CSF contamination. As a result, attempts to perform tractography of the fornix with the original tissue types failed. To overcome this limitation, we used subject-specific dilated white matter masks in which we constrained the tractography of the fornix.

**Table 1:** Anatomical ROIs used to define each white matter tract of interest. White matter tracts were defined as the streamlines connecting a seed and a target ROI. Appropriate exclusion ROIs were used to eliminate unwanted streamlines that did not belong to the studied tract.

Tract	Seed ROI	Target ROI	Exclusion ROIs
Uncinate fasciculus	Temporal pole	Ventromedial prefrontal cortex	Contralateral ventromedial prefrontal cortex; temporal pole; amygdala
Dorsal cingulum bundle	Cingulate isthmus	Ventromedial prefrontal cortex	Parahippocampal cortex; lingual gyrus; controlateral ventromedial prefrontal cortex; and posterior cingulate cortex
Ventral cingulum bundle	Parahippocampal cortex	Cingulate isthmus	Mask of the ipsilateral uncinate fasciculus; controlateral posterior cingulate cortex; and cingulate isthmus
Fornix	Mammillary bodies	Hippocampus (both hemispheres)	

Quality control of tract reconstructions was performed by visually inspecting each tract, superimposed on the matching T1w and gray and white matter masks. Reconstruction of the UF and the fornix failed for one subject. Therefore, these tracts were not included in the following analyses.

### Prefrontal–limbic tract microstructure

Prefrontal–limbic tract microstructure was assessed with several diffusion parameters: fractional anisotropy (FA), axial diffusivity (AD), and radial diffusivity (RD). Diffusion parameter maps were mapped onto tracts using a sampling scheme of 1000 points location for each streamline contained in the tract. We computed the within-streamline averages of each diffusion parameter, followed by between-streamline average of these within-streamline average values. We thus obtained tract-specific diffusion parameters describing tract microstructure.

### Statistical analyses

#### Age-related differences of memory performance and white matter microstructure

We preliminarily described the age-related differences of (i) EM scores and (ii) diffusion parameters describing white matter microstructure. This allowed us to verify if EM scores and diffusion parameters were indeed associated to age in order to further describe maturity profiles of each tract and their relation to the development of EM abilities. Age-related differences were examined with Pearson correlations between EM scores or diffusion parameters, and age. The *P*-values were adjusted for multiple comparisons with false discovery rate (FDR: Benjamini and Hochberg 1995).

#### Multivariate assessment of the relationship between white matter and memory performance

Our first aim was to examine the multivariate relationship between memory performance (short-delay free recall, long-delay free recall, long-delay cued recall) and diffusion parameters. We used PLSC, also known as PLS-SVD (for singular value decomposition), a dimensionality reduction technique commonly used in neuroimaging to examine structure–function relationships (e.g. Chen et al. 2019; Keresztes et al. 2017; Krishnan et al. 2011, 2011; Van Roon et al. 2014). PLSC aims at performing dimensionality reduction with SVD by finding the optimal representation of the shared information between two sets of variable (e.g. EM scores and diffusion parameters). It can thus be understood similarly to principal component analysis (PCA). PCA aims to find latent variables maximizing the variance shared by a set of variables X, while PLSC aims at finding latent variables maximizing the shared information (correlation) between two sets of variables X and Y.

We used PLSC to estimate the relationship between diffusion parameters describing tract microstructure (X) and EM scores (Y). We used PLSC models for each tract separately. Models included diffusion parameters from both hemispheres of a given tract (X) and the three EM scores (Y). PLSC works by decomposing the correlation matrix: corr(X,Y) with SVD. A latent variable LV is then estimated, which represents the optimal statistical association between the singular vectors of X and Y. The significance of the variance of the singular vectors explained by the latent variable was assessed with permutation testing (5,000 permutations). Additionally, the reliability of the contributions of the diffusion parameters with the latent variable was assessed with 5,000 bootstraps. This yields bootstrap ratios that represent the reliability of the contributions of the diffusion parameters to the multivariate association between diffusion parameters and EM. The weights of the EM scores were defined as the correlation between each EM score and the latent variable.

#### Multivariate representations of the microstructural maturity of white matter

Our second aim was to understand how the multivariate association between white matter microstructure and EM performance was related to age. In this order, we used PLSC to define latent variables representing, for each tract, the optimal statistical association between diffusion parameters and age. The same statistical principles as the ones described above (permutation testing, bootstraps) were used. Because the obtained latent variables maximize the shared information between diffusion parameters and age, the projections of individual subjects on the latent variable can be used to study interindividual differences of the shared information between tract microstructure and age (Keresztes et al. 2017). These individual projections can thus be interpreted as a subject-level “tract maturity score” of a given white matter tract (see Keresztes et al. (2017) for a similar approach on hippocampal maturation). Indeed, if the maturity scores are positively correlated with age, then the higher the value of an individual maturity score, the more it conveys white matter properties represented in older (compared to younger) children, and conversely. We examined the relationship between this statistical approximation of the microstructural maturity of white matter and EM performance by correlating tract-specific maturity scores with EM recall separately for each EM recall score. We verified the specificity of the relationship between EM recall and tract maturity by correlating tract maturity with a measurement of memory discrimination, which was also acquired in our behavioral assessment protocol and published in a previous study (Bouyeure et al. 2021). Raw *P*-values of EM scores/tract maturity scores were adjusted for multiple comparisons with FDR.

**Fig. 2:**
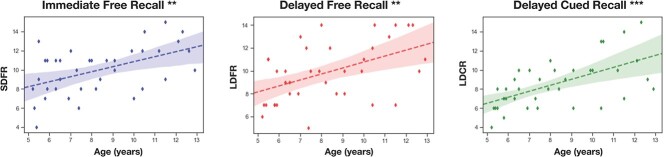
Plots of the regressions between memory scores and age. The shades represent the 95% confidence intervals. SDFR = short-delay free recall. LDFR = long-delay free recall. LDCR = long-delay free recall. ^*^^*^: *P* < 0.01; ^*^^*^^*^: *P* < 0.001 (corrected).

#### Effect of age on tract maturity–EM performance associations

Our third aim was to verify whether the associations between tract maturity and EM performance of aim 2 differed as a function of age. In this order, we plotted tract maturity scores–EM performance relationships separately for younger and older children. We used the age of 7 as a cutoff to define age groups because it roughly corresponds to the offset of childhood amnesia, and because it allowed an equivalent distribution of subjects into age groups. We statistically verified the influence of age groups by using linear regression models predicting EM scores with the tract maturity score of a given tract, age group, and the age group^*^tract maturity score interaction.

## Results

### Age-related differences of memory performance and white matter microstructure

We first described age-related differences of verbal EM scores. Immediate free recall was associated to age (*F* = 11.71, *R*2 = 0.251, *β* = 0.52, *P* < 0.01), as well as delayed free recall (*F* = 11.02, *R*2 = 0.239, *β* = 0.52, *P* < 0.01), and delayed cued recall (*F* = 18.98, *R*2 = 0.35, *β* = 0.626, *P* < 0.001), showing, in all cases, linear increases of verbal EM performance with respect to age. Figure 2 shows the relationships between EM scores and age. Adding sex as a covariate in these models did not change the significance of age and no significant sex differences were found for all EM scores.

**Fig. 3:**
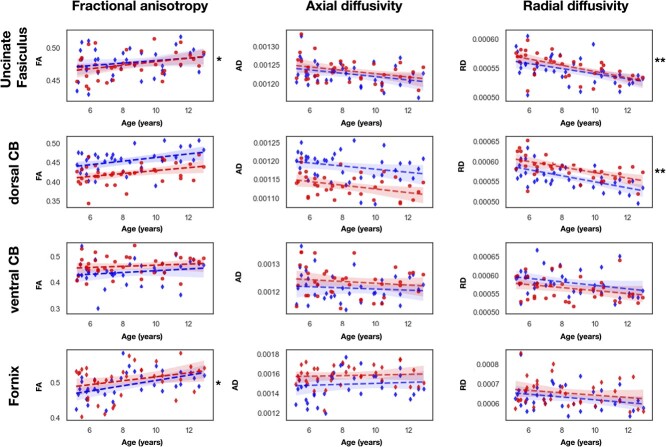
Plots of the regressions between diffusion parameters and age. The relation between diffusion parameters and age is represented for each tract. Red: Left hemisphere. Blue: Right hemisphere. The shades represent the 95% confidence intervals. CB = cingulum bundle. FA = fractional anisotropy. RD = radial diffusivity. AD = axial diffusivity. ^*^^*^: *P* < 0.01 (corrected).

We examined age-related differences of diffusion parameters with linear models in which diffusion parameters were the dependent variable and age the predictor variable. Figure 3 shows the plots of the linear regressions between age and diffusion parameters. The correlation coefficients and p-values are reported in Table 2. Among all diffusion parameters, the RD was the most correlated with age, showing a negative correlation. AD was never significantly correlated with age. The UF and the dorsal CB had RD and FA (for the right UF) significantly correlated with age. The FA of the left fornix was also significantly correlated with age (*P*-values are corrected with FDR).

**Table 2:** Pearson correlation values between age and diffusion parameters for each white matter tract. CB = cingulum bundle. FA = fractional anisotropy. RD = radial diffusivity. AD = axial diffusivity ^*^:*P* < 0.05; ^*^^*^:*P* < 0.01; ^*^^*^^*^:*P* < 0.005 (corrected).

Tract	Hemisphere	Diffusion parameter	*r*-value
Uncinate fasciculus	Left	FA	0.24
		RD	−0.46^*^^*^
		AD	−0.32
	Right	FA	0.35^*^
		RD	−0.61^*^^*^
		AD	−0.34
Dorsal CB	Left	FA	0.36
		RD	−0.61^*^^*^
		AD	−0.30
	Right	FA	0.33
		RD	−0.56^*^^*^
		AD	−0.38
Ventral CB	Left	FA	0.16
		RD	−0.31
		AD	−0.09
	Right	FA	0.14
		RD	−0.29
		AD	−0.11
Fornix	Left	FA	0.44^*^
		RD	−0.27
		AD	0.06
	Right	FA	0.28
		RD	−0.21
		AD	0.02

### Multivariate associations between episodic memory and white matter microstructure

Our first aim was to explore the multivariate relationship between EM scores (short-delay free recall, long-delay free recall, long-delay cued recall) on the one hand and the diffusion parameters of each tract (from both hemispheres) on the other hand. We used PLSC to extract latent variables representing the shared information between EM scores and diffusion parameters. The significance of the variance explained by the latent variable was assessed with 5,000 permutations of the data.

#### Uncinate fasciculus

**Fig. 4:**
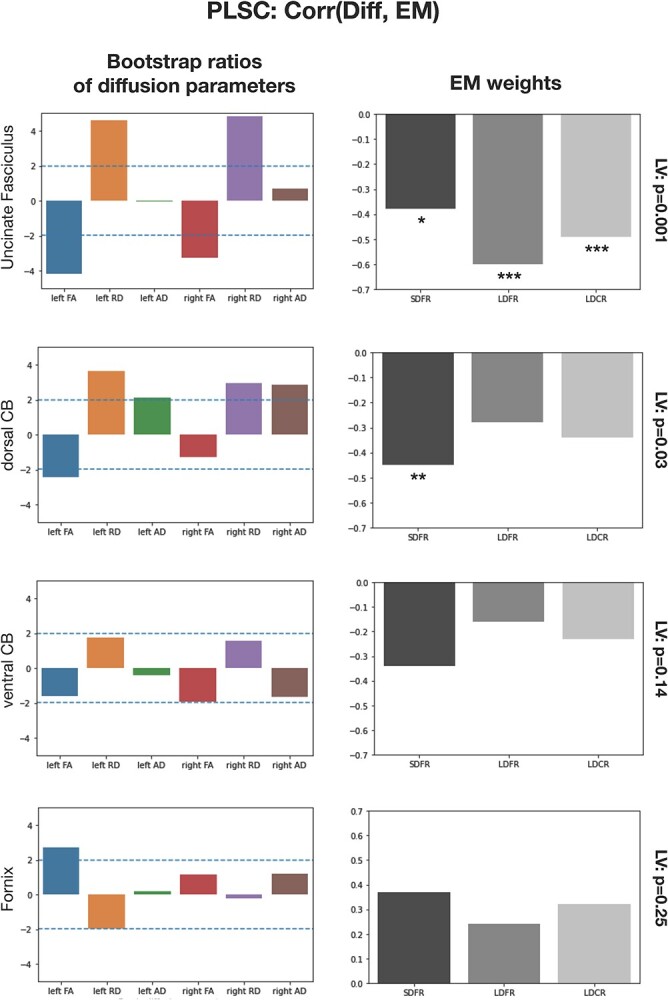
PLSC results extracting latent variables representing the shared information between diffusion parameters and EM scores. Left column: Bootstrap ratios of diffusion parameters showing the reliability of the contribution of each diffusion parameter to age. A ratio ±1.96 (blue dotted line) shows that the contribution of the diffusion parameter to the latent variable is reliable. Right column: Weights (correlation values) of EM scores on the obtained latent variable. The significance of each LV is shown on the top right. SDFR = short-delay free recall. LDFR = long-delay free recall. LDCR = long-delay free recall. ^*^:*P* < 0.05; ^*^^*^:*P* < 0.01; ^*^^*^^*^:*P* < 0.005.

A latent variable significantly represented the multivariate association between EM scores and diffusion parameters of the left and right UF (*P* = 0.001). Bootstrap ratios indicated that FA and RD diffusion parameters (both hemispheres) were reliable components contributing to the latent variable (values above +1.96 or below −1.96), but not left or right AD. Bootstrap ratios of FA and RD were of opposite signs given that they respectively correlate positively and negatively with cognitive function. In this case, RD bootstrap ratios were positive and FA bootstrap ratios were negative, but these signs only show that diffusion parameters contribute to the latent variable in opposite direction and could be arbitrarily inverted. Still, the weights of the EM scores on the latent variable were negative correlations given that RD was positively loaded on the latent variable and FA negatively. Long-delay free recall was the variable with the most important weight on the latent variable (*r* = −0.60, *P* = 0.0001), followed by long-delay cued recall (*r* = −0.49, *P* = 0.002) and short-delay free recall (*r* = −0.38, *P* = 0.02). Bootstrap ratios of diffusion parameters and weights of EM scores on the latent variable are shown in Fig. 4.

#### Dorsal cingulum bundle

A latent variable significantly represented the multivariate association between EM scores and diffusion parameters of the left and right dorsal CB (*P* = 0.03). All diffusion parameters except right hemisphere FA were reliable components contributing to the latent variable. Left and right RD were the most reliable contributors. The weights of EM scores showed that short-delay free recall was significantly represented in the latent variable (*r* = −0.45, *P* = 0.006) as well as long-delay cued recall (*r* = −0.35, *P* = 0.03). Long-delay free recall was not significantly loaded on the latent variable (*r* = −0.28, *P* = 0.09) (Fig. 4).

#### Ventral cingulum bundle

We did not find a latent variable significantly representing the association between EM scores and the diffusion parameters of left and right ventral CB (*P* = 0.14). This suggests that the microstructure of left and right ventral CB was not associated to measures of EM recall in a multivariate fashion.

#### Fornix

We did not find a latent variable significantly representing the association between EM scores and the diffusion parameters of left and right fornix CB (*P* = 0.25).

Thus, there were latent variables significantly representing the relationship between the microstructure of the UF and the dorsal CB with measures of EM recall in children, but no multivariate relationships were found for the ventral CB and the fornix.

### Multivariate associations between white matter microstructure and age: tract maturity scores

Our second aim was to examine if the multivariate associations reported in the previous sections could also be described in terms of a relation between individual differences of tract maturity and individual differences of EM performance. In this aim, we extracted multidimensional representations of the shared information between tract microstructure and age with PLSC (“tract maturity scores”), which we then correlated to EM.

#### Uncinate fasciculus

**Fig. 5:**
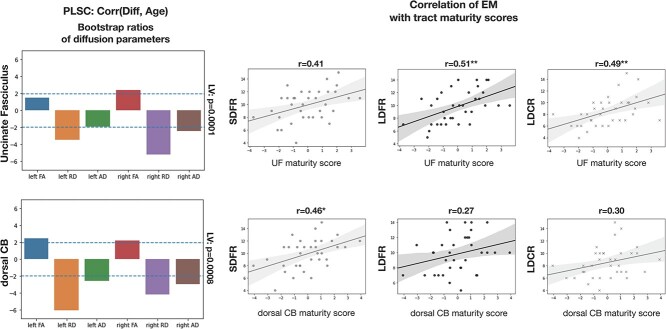
Tract maturity scores for the UF and the dorsal CB obtained from PLSC, and correlation between tract maturity scores and EM. Left: Bootstrap ratios of the diffusion parameters showing the reliability of their contribution to the latent variables. Right: Regression plots showing the relation between tract maturity scores and EM performance. SDFR = short-delay free recall. LDFR = long-delay free recall. LDCR = long-delay free recall. ^*^^*^^*^:*P* < 0.005 (corrected with FDR).

We extracted a single latent variable significantly representing the association between age and the diffusion parameters of left and right UF (*P* = 0.001). The correlation between this maturity profile and age was of −0.61. Bootstrap ratios indicated that all diffusion parameters except left FA were reliable components contributing to the maturity score, with left and right RD being the most reliable. Because RD is the most reliable component of the maturity profile and since RD decreases with age, this explains why the maturity profile is negatively correlated with age. We multiplied the maturity score by −1 in order to have a positive correlation between the maturity score and age (the higher the maturity score, the older the children) rather than the opposite. This was arbitrary and for interpretative purposes. Figure 5 shows the correlation with age of the (inverted) maturity score as well as the bootstrap ratios of the diffusion parameters.

#### Dorsal cingulum bundle

A single latent variable significantly represented the association between age and the diffusion parameters of the left and right dorsal CB (*P* = 0.0008, 5,000 permutation tests). The correlation between this maturity profile and age was of −0.65. We hence also inverted the sign of these maturity scores. Bootstrap ratios indicated that all diffusion parameters were reliable components contributing to the maturity profile, with left and right RD being the most reliable components (Fig. 5).

We also examined if the fornix and the ventral CB were associated to age in a multivariate fashion. We did not find latent variables significantly representing the variance shared by the diffusion parameters of these tracts and age (fornix: *P* = 0.07; ventral CB: *P* = 0.25; 5,000 permutations). This was expected given the few correlations between the diffusion parameters of these tracts and age (Fig. 3).

#### Relation between maturity scores and EM recall

We examined the relationships between tract maturity scores and EM recall scores. The maturity score of the UF was not correlated with short-delay free recall (r = 0.41, corrected *P* = 0.06). It was however correlated with long-delay free recall (*r* = 0.51, corrected *P* = 0.01) and long-delay cued recall (*r* = 0.49, corrected *P* = 0.01) (Fig. 5). Thus besides a global multivariate association between diffusion parameters of the UF and EM scores we also showed that the maturity of the UF (defined by a latent variable representing shared information between UF diffusion parameters and age) correlated with EM. The maturity score of the dorsal CB was correlated with short-delay free recall (*r* = 0.46, corrected *P* = 0.03), but not with long-delay free recall (*r* = 0.27, corrected *P* = 0.20), or with long-delay cued recall (*r* = 0.30, corrected *P* = 0.20). The specificity of the correlation between the maturity of the dorsal CB and short-delay free recall is coherent with the fact that this EM score was the main weight on the multivariate association between dorsal CB diffusion parameters and EM scores (Fig. 4). The evidential value of the significant correlations was further assessed by estimating Bayes factors (BF10) from the significant correlations. BF10 indicates the likelihood of the tested hypotheses relatively to the null. We found BF10 of 8.51 (correlation between CB maturity score and short-delay free recall), 24.6 (correlation between UF maturity score and long-delay free recall), and 15.3 (correlation between UF maturity score and long-delay free recall). This indicates moderate evidential value (3 > BF10 > 10) to decisive evidential value (BF10 > 10 or BF10 > 20).

We further examined if the relationship between tract maturity score and EM recall was specific to EM recall by also correlating tract maturity profiles with measurements of memory discrimination, a mnemonic process that is often specifically associated with the maturation of hippocampal subfields (Keresztes et al. 2017). Memory discrimination was not correlated with tract maturity scores of the UF (*r* = 0.22, corrected *P* = 0.24) or of the dorsal CB (*r* = 0.35, corrected *P* = 0.20).

### Influence of age on tract maturity–EM recall relationship

**Fig. 6:**
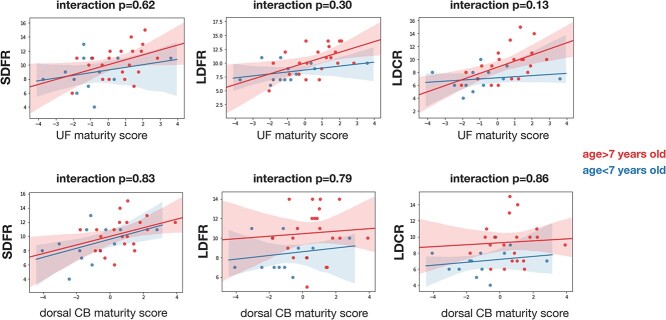
Interaction plots showing the relations between tract maturity scores and EM recall as a function of age groups. Children younger than 7 years old are plotted in blue and children older than 7 years old in red. SDFR = short-delay free recall. LDFR = long-delay free recall. LDCR = long-delay free recall.

Our third aim was to examine whether tract maturity–EM relationships differed as a function of age. As the dorsal CB and the UF have a protracted maturation, it is possible that the maturity score of these tracts contribute differently to EM performance in older children compared to younger children. While the correlation between tract maturity and EM was more important in older children in a few comparisons, no interaction terms between tract maturity and age groups were significantly associated to EM scores (Fig. 6).

## Discussion

Our aim was to study age differences in the microstructure of prefrontal–limbic tracts involved in EM recall, as well as the relation between their microstructure and EM recall performance, in children aged from 4 to 12 years old. The main results were as follows: (i) UF and dorsal CB diffusion parameters were associated with EM recall scores in a multivariate perspective. The ventral CB and the fornix were not associated with EM recall. (ii) there was multivariate associations between UF and dorsal CB diffusion parameters and age, yielding within-subject tract maturity scores. The tract maturity score of the UF was correlated with long-delay conditions of EM recall. Tract maturity score of the dorsal CB was only correlated with short-delay free recall. (iii) Relationships between tract maturity and EM did not differ as a function of age.

### UF and dorsal CB microstructure are associated with EM recall

Our first aim was to analyze the relationship between tract microstructure and EM performance. We demonstrated that there was a multidimensional pattern relating differences of UF and dorsal CB microstructure with EM abilities in the developing brain (Fig. 4). Therefore, for the UF and the dorsal CB, but not the ventral CB and the fornix, the relationship between their diffusion parameters and EM scores could be described with single latent variables representing their shared information.

Because of this multivariate approach, aspects of all diffusion parameters and EM scores were taken into account to extract significant latent variables picturing a global relationships. With this in mind, analysis of the contributions of diffusion parameters and weights of EM scores showed differences in the structure of these latent variables. For the UF, the diffusion parameters contributing reliably to the latent variable were FA and RD, but not AD. All EM scores were significantly correlated with the latent variable (negative correlations). EM scores were negatively correlated to the latent variable. The contributions of diffusion parameters assessed with bootstrap ratios showed that the contribution of RD was positive and the contribution of FA negative. This suggests that aspects of EM performance were positively related to FA values and negatively related to RD values, albeit in a multivariate fashion. For the dorsal CB, FA, AD, and RD (with the exception of right FA) were reliable contributors to the multivariate association with EM scores. Short-delay free recall was the only EM score that significantly contributed to the latent variable, which is coherent with the fact that the tract maturity profile of the dorsal CB was correlated with short-delay free recall but not with other EM scores. There was more important contributions of AD for the multivariate association with the dorsal CB compared to the UF, and the association for the dorsal CB was more correlated with short-delay free recall than other scores. Hence, the dorsal CB and the UF were related to EM scores in distinct ways.

### Maturity scores of the UF and the dorsal CB are associated to EM recall

Our second aim was to determine if the multivariate relationship between tract microstructure and EM performance could be further described in terms of a relationship between tract maturity and EM.

For the UF and the dorsal CB, we found latent variables significantly representing their relationship with age. This is not surprising given that these two tracts are known to have a particularly protracted maturation compared to other white matter tracts (e.g. Lebel et al. 2008, 2012; Simmonds et al. 2014; Reynolds et al. 2019). Their extended maturation is likely related to the maturation of some of the cortical regions connected by these tracts, e.g. the prefrontal and posterior parietal areas (Sowell et al. 2004; Lebel et al. 2008; Dosenbach et al. 2010; Arain et al. 2013; Caballero et al. 2016). For the fornix and ventral CB, however, we did not find significant associations between their microstructure and age. These PLSC results are overall in line with diffusion parameters–age bivariate correlations (Fig. 3). The fornix has been described as an early-maturing tract (Dubois et al. 2008, 2012; Lebel et al. 2008, 2012). The maturation of the ventral CB is poorly known, but the only one developmental study (to our knowledge) that separated dorsal ventral CB segments showed an earlier maturation of the ventral segment (Simmonds et al. 2014). Compared to the dorsal CB, the earlier maturation of the ventral CB could be explained by the fact that it mainly connects limbic regions, which have an earlier maturation than the prefrontal and posterior parietal regions connected by the dorsal segment (Utsunomiya et al. 1999; Paus 2005; Arain et al. 2013).

The analysis of the weights of EM scores on the latent variables from our initial PLSC analyses showed that EM scores were differently related to the UF and the CB. We thus examined the relationships between tract maturity scores and EM recall scores separately. We found significant correlations between tract maturity scores and EM recall. Specifically, the maturity score of the UF was correlated to long-delay conditions of EM recall (free and cued). The maturity of the dorsal CB was only correlated to short-delay recall (Fig. 5). These results are in line with the EM weights of our initial multivariate analyses, which yielded similar observations (Fig. 4). This suggests a specialization of structure–function relationships between white matter tracts and distinct aspects of EM recall in children. More precisely, white matter microstructural differences related to age, thereby expressing tract maturity, were related to EM recall differently for the UF and the dorsal CB. These two tracts could hence contribute to distinct aspects of EM function and development.

The relationship between UF microstructure and EM recall was reported by previous studies on older children and adolescents (Mabbott et al. 2009; Schaeffer et al. 2014; Samara et al. 2019). We develop these findings by showing that distinct aspects of delayed EM recall were associated to a multivariate representation of the microstructural maturity of the UF. Some diffusion parameters contributed more to the UF maturity score than others (e.g. RD). However, age-related differences of all diffusion parameters importantly or marginally contributed to the maturity score. Hence age-related differences of microstructural properties typically inferred from diffusion parameters, such as myelination, axonal density, and overall tissue integrity, are likely all related to individual differences of EM performance.

Retrieval of episodic memories following a delay has been shown to elicit activity in the hippocampal and medial temporal lobe regions and in the prefrontal cortex (e.g. Dupont et al. 2001; Maril et al. 2010; Hayama et al. 2012; Rugg et al. 2015). These areas are precisely the ones connected by the UF (Olson et al. 2015). A higher tract maturity score of the UF could thus be associated with microstructural differences causing a quicker and more efficient transmission of information between the prefrontal and medial temporal regions. This could in turn benefit the development of the capacity to recall episodic information following delays.

Contrary to the UF, the maturity score of the dorsal CB was specifically correlated to short-delay free recall. To our knowledge, this is the first time that a relationship between dorsal CB microstructure and EM recall is reported in children. In the CVLT-c, short-delay free recall is administered immediately after learning a distractor list. Success at this task thus likely depends on the ability to efficiently retrieve items that were learned moments ago while inhibiting items of the distractor list. This could involve mnemonic control and executive function–related processes. Accordingly, the dorsal CB has been associated with such cognitive functions by previous studies in adults (Wendelken et al. 2015; Bubb et al. 2018). Short-delay free recall performance could be related to the maturity of the dorsal CB, which would ensure more efficient information transmission between regions essential for mnemonic control and executive functions, such as the dorsolateral prefrontal cortex, anterior cingulate cortex, and parietal areas.

We provide evidence regarding the specificity of our findings given that tract maturity scores of the UF and the dorsal CB were correlated with distinct, nonoverlapping EM scores, and both maturity profiles did not correlate with memory discrimination, which has been shown to be associated to a multivariate expression of hippocampal maturity (Keresztes et al. 2017).

### Similar tract maturity score–EM performance in younger and older children

Contrary to our hypothesis, the relationship between maturity scores and EM performance did not differ as a function of age (Fig. 6). A previous study examining the white matter correlates of EM in 4 and 6 years old (Ngo et al. 2017) found no relation between the UF or between hippocampal–prefrontal connectivity and EM recall. The authors speculated that this absence of association could be related to the particularly protracted maturation of prefrontal–hippocampal connectivity, e.g. because the medial prefrontal cortex and the UF are not mature enough, during early childhood, to be fully involved in these cognitive processes. Our results rather show that the microstructural maturity of prefrontal tracts with a protracted maturation contributes to EM recall similarly in younger and older children. Methodological differences and the fact that our studied age span (despite including 4–6 years old children) is overall older could account for these differences. The present results nevertheless suggest that the UF and the dorsal CB might play an earlier role in EM function during development than previously thought.

### Limitations and future directions

Our study has several limitations. The main one is that we aimed to characterize tract maturity with a multivariate representation of the association between tract microstructure and age while using a cross-sectional design. Descriptions of tract “maturity scores” are hence only statistical approximations, which do not directly describe developmental dynamics, contrary to longitudinal designs (e.g. Giorgio et al. 2010; Simmonds et al. 2014; Krogsrud et al. 2016). Contrasting the contributions of white matter maturation to EM development would benefit from a longitudinal design that investigates the relation between tract maturation and cognitive competence over developmental time. A related limitation is that this design prevents us from trying to analyze causal relations between tract maturity and EM performance. Therefore, while we showed a relationship between a statistical representation of tract maturity and EM performance, other causes could account for this relationship. Such factors could include the maturation of other white matter tracts, cortical maturation, or the development of other aspects of EM or of other cognitive functions. We however provide some evidence of the specificity of the reported associations.

Another limitation is that our sample size was small, particularly compared to the important age range studied (4–12 years of age). The acquisition of neuroimaging data can be particularly challenging in children and sample size is a limitation often faced by developmental studies. Studying larger samples restricted to smaller age groups could lead to more specific investigation of the contribution of white matter maturity to EM development.

Third, we used multishell high resolution with state-of-the-art methods for modeling crossing/kissing fibers. Still, tract microstructure was assessed by diffusion parameters that depend on tensor modeling, a technique known to have a number of methodological limitations. Past years have seen a surge of methodological development that take benefit from multishell data to estimate fiber microstructure in a way that overcomes some of the traditional limitations associated to tensor estimation, such as estimation of neurite orientation dispersion and density imaging (NODDI), or fiber density and cross-section (Raffelt et al. 2012, 2017). To date, no study investigated memory development using these recent diffusion MRI data analysis techniques.

Fourth, we only focused on a narrow aspect of EM function, i.e. recall tasks from the CVLT-c. Studying the white matter correlates of other EM processes, such as relational memory, context memory, or pattern separation/completion, might provide insightful results regarding memory development, especially in younger populations (see, for instance, Ngo et al. 2017, 2018, 2019). Future studies will benefit from investigating the white matter correlates of a variety of EM processes during development, a topic that has little been studied to this day.

Finally, we did not find relations between ventral CB and fornix microstructure and EM performance, despite the fact that these tracts, particularly the fornix, have been repeatedly associated to EM function in adults. An interesting avenue of research is thus the further investigation of the role of these tracts in EM development. The microstructure of the fornix has been associated with relational memory and context memory (Hodgetts et al. 2017; Schwarb et al. 2019) and with verbal EM recall in the context of normal aging and mild cognitive impairments (Metzler-Baddeley et al. 2011). The ventral CB has mainly been associated to object recognition, discrimination, and spatial navigation in animal models (Bubb et al. 2018) and to EM performance in pathological contexts in human adults (Metzler-Baddeley, Hunt, et al. 2012; Metzler-Baddeley, Jones, et al. 2012). The cognitive demands of EM recall tasks used here could hence not rely on the fornix and the ventral CB in the context of healthy development. This hypothesis is purely speculative given that the relation of these two tracts to EM have been understudied in children.

## Conclusion

We described multivariate associations between the microstructure of the UF and the dorsal CB and EM recall. These associations could be described in terms of interindividual differences of tract maturity. The progressive maturation of these two tracts is therefore likely to contribute to the gradual unfolding of EM function during childhood. Furthermore, these tracts were associated to distinct aspects of EM function. Our results also suggest that these two tracts could be involved in EM function early during development. This calls for further research to determine more precisely the contribution of white matter microstructure on EM development and function.
